# Spatial distribution and prevalence of physical disabilities in the provinces of Iran

**Published:** 2015

**Authors:** H Abbastabar, A Alizadeh, M Darparesh, S Mohseni, N Roozbeh

**Affiliations:** * Islamshahr Health and Treatment Network, Department of Health, Tehran University of Medical Sciences, Tehran, Iran; ** Department of Public Health, Faculty of Health, Hormozgan University of Medical Sciences, Bandar Abbas, Iran; *** Research Center for Social Determinants in Health Promotion, Department of Research and Technology, Hormozgan University of Medical Sciences, Bandar-e-Abbas, Iran; **** Department of Public Health, Faculty of Health, Hormozgan University of Medical Sciences, Bandar Abbas, Iran; ***** Reproductive Health, Shahid Beheshti University of Medical Science, Tehran, Iran

**Keywords:** physical disabilities, spatial distribution, Iran

## Abstract

**Introduction:** To identify the influential social factors and spatial distribution of physical disabilities in Iran between 2006 and 2011.

**Materials and Methods:** First, the prevalence of physical disability in each province between 2006 and 2011 was mapped via GIS. Moreover, the percentage of physical disability was estimated with regard to age, sex, and residential area. Finally, the prevalence of physical disability was estimated with regard to the afore-mentioned variables.

**Findings:** The findings revealed that in the majority of the provinces of Iran, there was a decreasing trend in the prevalence of physical disability from 2006 to 2011. The prevalence of physical disability in the total population of the country was also decreased during these years. The results were also indicative of a higher prevalence among men than among women and also in rural residential areas than in urban areas.

**Conclusion:** The results of this research can be used to identify the high- and low-risk areas. In addition, this information can be used for the etiology and the specification of the factors that cause the residents of some regions to get afflicted more than the others.

## Introduction

Since the beginning of his life, the human being has been faced with various diseases, accidents, and hazardous events. These have constantly threatened one’s life and health and have led to different types of physical/ kinetic disabilities.

The physical disability refers to the limitations of the physical performance, movement, skill, or ability of an individual. It includes cases that disable people from carrying out their daily routine activities [**1**]. Physical disability can also be visualized as losing part or all of one’s body performance (e.g. walking, control over urine, and so on). It can also refer to the loss of one part of the body. In terms of the underlying causes, there are two types of physical disabilities: firstly, the prenatal physical disabilities, which exist from the time of birth throughout one’s life. They could be due to inadequate provision of oxygen, the inspiratory system blockage, brain injury at the time of birth and premature delivery. The second type is the postnatal disabilities, which occur after the birth of the child. The underlying causes could be accidents, infection, or other diseases [**2**].

Statistics show that about 2 million people who have lost parts of their body live in the U.S.A. [**3**]. Moreover, about 18500 amputation cases occur in the U.S. [**4**]. The probability of amputation among African Americans is 4 times as frequent as the white population [**5**]. The most common reasons for losing part of one’s body are vascular diseases including diabetes along with arterial/ environmental diseases (54%), trauma (45%) and cancer (less than 2%) [**3**]. About half of the people whose amputation was due to vascular diseases lose their life within 5 years. This has been larger than the frequency of the loss within 5 years for breast cancer, colon cancer and prostate cancer [**6**]. Among diabetic patients who have had amputation of the lower part of their body, over 55% will be in need of amputation of their other leg within the next 2-3 years [**7**].

In 1986, there were 288508 physically/ kinetically disabled (199505 men and 89003 women) in Iran. They comprised 63.7% of the total disabled population. The frequency of different types of disability was leg, arm, leg and arm amputation or both of them respectively 188402, 89135 and 10971. Accidents and diseases accounted for 49.4 and 31.3 percent of physical/ kinetic disability among men, while accidents and diseases accounted for the main causes of such disabilities among women. In 2011, the population of the physically/ kinetically disabled grew and reached 601886 (391207 men and 210679 women). However, the ratio of the physically/ kinetically disabled compared to the total number of the disabled in the country decreased from 63.7% to 47.4% [**8**]. In 2009, the hospitalization costs of amputation exceeded 8.3 billion dollars [**9**].

Besides threatening one’s health, physical disability affects the mental and social health of the young to a great extent [**10**]. Among the side effects of amputation are body shape transformation, mood, movement, sexual matters, career-oriented activities, and self-care abilities [**11**-**14**]. The occurrence and prevalence of physical/ kinetic disabilities in societies is highly dependent on the load of diseases, industrialization, behavior, and culture of people. No comprehensive research has been conducted so far in Iran to determine the distribution of these disabilities. Such knowledge can be the first step in managing and organizing the control of the disease. Therefore, the present research seeks to identify the spatial distribution of physical disability in Iran between 2006 and 2011.

## Materials and Methods

This descriptive research was carried out with the aim of determining the spatial distribution and estimating the physical disabilities in the provinces of Iran. The data related to the whole population used in this study were obtained from the national statistics center. The data concerning the physical disability were obtained from a welfare organization and also the national statistics center of Iran. The physical disability in this study refers to the loss of part of the body such as a leg or an arm, amputation, anatomic disorder of the body and also performance disorders.

Briefly, disabled persons or their parents refer to welfare organization and fill out an application form. Then they will be investigated in a medical commission. If the commission verified their disabilities, they would be divided into mild, moderate, severe, and greatly severe groups.

Firstly, the prevalence of the disease in every province was mapped via GIS in 2006 and 2011. To estimate the prevalence of the physical disability in each province during these years, the frequency of that physical disability, in that year, was divided by the total population of that province during that same year. Through this procedure, high- and low-risk areas can be identified.

Moreover, in this study, the percentage of physical disabilities was reported with regard to age, sex, and residential area. To do this, all the participants were first divided into the following age groups: 0-14, 15-29, 30-44, 45-59, 60-74, 75 plus. In terms of residential area they were divided into urban and rural areas and as for sex, two groups of male, and female were considered. Subsequently, the frequency of physical disability in each sub-group was divided by the total population of that group. To estimate the percentage, the total number of the disabilities in each sub-group (sum of the columns) was divided by the total number of physical disabilities (sum). All the calculations were done by using Microsoft Office Excel 2007. To do the mapping, ArcMap 9.3 GIS software by ESRI was employed.

## Results

**Fig. 1:**
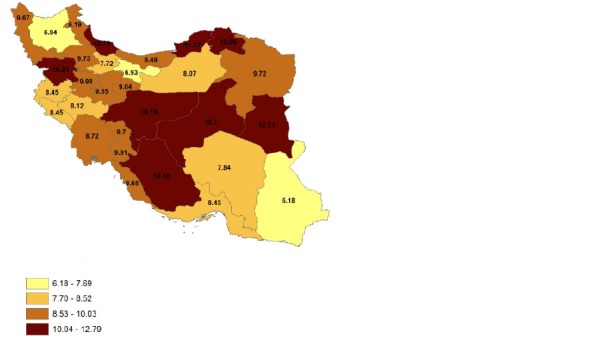
Prevalence of physical impairment during 2006 in the provinces of Iran

**Fig. 2:**
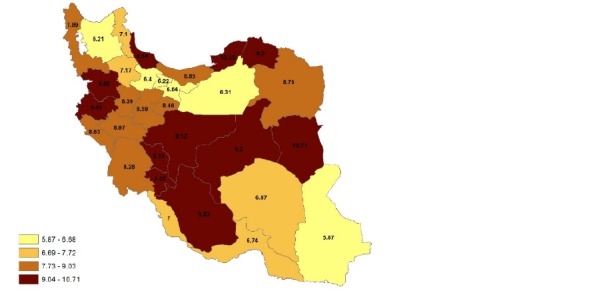
Prevalence of physical impairment during 2011 in the provinces of Iran

**Fig. 1** and **Fig. 2** indicated the prevalence values of physical disability in all the provinces during 2006 and 2011. **Fig. 1** indicated that in 2006, physical disabilities were the most prevalent in Gilan province and the least prevalent in Sistan and Blochestan province. Similarly, **Fig. 2** showed that in 2011, the highest prevalence of physical disabilities was in south Khorasan province and the least prevalence was in Sistan and Blochestan province. The two findings indicated an increasing trend during this time span.

**Table 1** is indicative of the percentage of physical disabilities between 2006 and 2011 with regard to age, sex, and residential area. The results presented in **Table 1 A** showed that in 2006, the highest percentage of physical disabilities among urban and rural men was 22.96 and 27.04 respectively that occurred in the age groups of 30-44 and 15-29. This percentage, among urban and rural women, was 20.25 and 21.83 and occurred in the age group of 15-29. Among the total cases of physical disability, the highest value (23.7%) belonged to the 15-29 age group. From the total cases of physical disability, 41.94% occurred among urban men, 24.24% among rural men, 21.02% among urban women, and 12.79% among rural women. The results presented in **Table 1 B** indicated that the highest values among urban and rural men were 27.63% and 26.5% occurring in the age group 15-29. Among urban and rural women, these values were 19.67% and 20.89%, which occurred in the age groups of 30-44 and 15-29. Out of the total cases of physical disabilities with regard to age, the highest value was 24.62%, which occurred in the age group 30-44. As for sex and residential area, 42.82% of the total cases occurred among urban men, 22.18% among rural men, 22.97% among urban women and 12.04 among rural women.

**Table 1:** Proportion of physical disorder in subgroups of age, sex, and residence location during 2006 and 2011

**Table 1 A**. Proportion of physical disorder in subgroups of age, sex and residence location during 2006										
	Male				Female				Total	
	Urban		Rural		Urban		Rural			
Age groups	Number	Percent	Number	Percent	Number	Percent	Number	Percent	Number	Percent
0 - 14	21002	8.04	15726	10.41	14695	11.22	11028	13.84	62451	10.02
15-29	62879	24.06	40836	27.04	26525	20.25	17398	21.83	147638	23.7
30-44	78294	29.96	35283	23.36	21999	16.8	11858	14.88	147434	23.67
45-59	46780	17.9	21875	14.48	18204	13.9	10520	13.2	97379	15.63
60-74	31172	11.93	20853	13.81	25774	19.68	15959	20.03	93758	15.05
75 <	21178	8.1	16447	10.89	23779	18.16	12920	16.21	74324	11.93
Total	261305	41.94	151020	24.24	130976	21.02	79683	12.79	622984	100
**Table 1 B ** Proportion of physical disorder in subgroups of age, sex and residence location during 2011										
	Male				Female				Total	
	Urban		Rural		Urban		Rural			
Age groups	Number	Percent	Number	Percent	Number	Percent	Number	Percent	Number	Percent
0 - 14	19583	7.61	12814	9.61	14501	10.5	9145	12.64	56043	9.32
15-29	54164	21.04	33170	24.87	25382	18.37	15118	20.89	127834	21.26
30-44	71135	27.63	35332	26.5	27170	19.67	14413	19.91	148050	24.62
45-59	64315	24.98	24020	18.01	21836	15.81	11056	15.28	121227	20.16
60-74	28126	10.92	14268	10.7	25718	18.62	11513	15.91	79625	13.24
75 <	20169	7.83	13745	10.31	23527	17.03	11128	15.38	68569	11.4
Total	257492	42.82	133349	22.18	138134	22.97	72373	12.04	601348	100

**Table 2** represents the prevalence of physical disabilities in the sub-groups of age, sex, and residential area between 2006 and 2011 per thousand people. **Table 2 A** shows this prevalence in 2006. The highest prevalence values among urban and rural men were 50.28 and 57.61 per thousand people occurring in the age range of 75 plus. The highest prevalence among urban and rural women was 57.86 and 58.58 per thousand people, again occurring in the age range of 75 plus. The last column of **Table 2 A** shows the prevalence of physical disabilities with regard to age. The highest value was 55.54 per thousand people, which occurred in the age group of above 75 years. The last row of **Table 2 A** shows the prevalence of physical disability with regard to sex and residential area. The prevalence value was estimated to be 10.63 among urban men, 13.44 among rural men, 5.53 among urban women, and 7.31 among rural women. Besides that, the prevalence of physical disabilities in the whole population of Iran was estimated to be 8.85 per thousand people. **Table 2 B** is indicative of the prevalence values reported for 2011. The highest values among urban and rural men were 34.38 and 39.29 per thousand people respectively occurring in the age group of 75 plus. The highest values among urban and rural women were 38.96 and 38.16 per thousand people ageing occurring at the age above 75. The last column of **Table 2 B** shows the prevalence of physical disabilities with regard to age. The highest prevalence value was 37.43 which occurred at the age group of 75 plus. The last row of **Table 2 B** indicates the prevalence of physical disabilities with regard to sex and residential area. It was estimated to be 9.54 among urban men, 12.29 per thousand among rural men, 5.19 among urban women and 6.83 per thousand among rural women. Furthermore, the prevalence value of physical disabilities in the whole population of Iran was found to be 8.01 per thousand people.

**Table 2:** Prevalence of physical disorders in 1000 persons by age, sex, and residence location during 2006 and 2011

**Table 2 A**. Prevalence of physical disorders in 1000 persons by age, sex, and residence location during 2006										
	Male				Female				Total	
	Urban		Rural		Urban		Rural			
Age groups	Number	Prev*	Number	Prev	Number	Prev	Number	Prev	Number	Prev
0 - 14	21002	3.58	15726	4.94	14695	2.63	11028	3.65	62451	1.17
15-29	62879	7.31	40836	10.22	26525	3.12	17398	4.56	147638	5.92
30-44	78294	14.47	35283	17.55	21999	11.11	11858	5.99	147434	10.13
45-59	46780	15.52	21875	20.22	18204	6.38	10520	8.74	97379	18.37
60-74	31172	24.62	20853	30.77	25774	21.81	15959	24.53	93758	24.83
> 75	21178	50.28	16447	57.61	23779	57.86	12920	58.58	74324	55.54
Total	261305	10.63	151020	13.44	130976	5.53	79683	7.31	622984	8.85
**Table 2 B ** Prevalence of physical disorder in 1000 persons by age, sex, and residence location during 2011										
	Male				Female				Total	
	Urban		Rural		Urban		Rural			
Age groups	Number	Prev	Number	Prev	Number	Prev	Number	Prev	Number	Prev
0 - 14	19583	3.21	12814	4.47	14501	2.48	9145	3.35	56043	3.19
15-29	54164	6.48	33170	9.39	25382	3	15118	4.54	127834	5.4
30-44	71135	10.86	35332	15.4	27170	4.27	14413	6.46	148050	8.49
45-59	64315	16.48	24020	19.79	21836	5.75	11056	8.4	121227	11.85
60-74	28126	18.91	14268	23.92	25718	16.55	11513	16.79	79625	18.42
75 <	20169	34.38	13745	39.29	23527	38.96	11128	38.16	68569	37.43
Total	257492	9.54	133349	12.29	138134	5.19	72373	6.83	601348	8.01
Prev*: Prevalence										

## Discussion

The findings of the present research revealed that in the majority of the provinces of Iran, the prevalence of physical disability decreased from 2006 to 2011. Moreover, the prevalence of physical disability in the whole population of Iran also decreased from 2006 to 2011. In addition, the findings were indicative of a higher occurrence of physical disabilities among men than among women and in rural areas compared to the urban.

According to the results in proportions, the highest percentage both among men and among women occurred in the age group of 15-45 (15-29 and 30-44). However, the results in prevalence showed that the highest values both among men and women occurred in the age group of 75 plus. In a study concerned with the frequency of amputation caused by electric shock among patients of Shahid Motahari hospital of Tehran conducted in 2006, 75% of the amputations was found to have occurred in the age group of 10-40 [**15**]. In another study on the categorized sex and age related distribution of physical disabilities by Ethgen et al. [**16**], the prevalence showed an increasing trend both among males and among females in the age range of 25-34. The average prevalence was found to be in the age group 25-34 and the highest prevalence occurred between 65 and 74 years of age [**16**]. In our study, the highest percentage of physical disability was observed in the age group of 15-45. However, the highest percentage was found among those of above 75 years of age. About this divergence, we could say that the denominators of the two fractions of these two indices were different. First, the denominator of the fraction of proportion was the total number of physical disability in the given group. The denominator of the fraction of the prevalence index was the total population of that given group. Secondly, the highest size of population belonged to the age group 15-45. This would cause that a higher number of individuals was exposed to the occurrence of physical disability. As a result, the proportion grew in size. On the other hand, since the denominator of the fraction, which is the prevalence of the population, also grew, the size of the prevalence of this age group did not grow significantly. With regard to the occurrence of the highest prevalence in the age group of 75 plus, it could be said that: firstly, many physical disabilities are not deadly. People afflicted with them live for a long time and reach the higher age groups. They also got involved in the estimations of the prevalence in the higher age groups, this way. Secondly, as mentioned earlier, the denominator is the prevalence in the population of the given age group and is usually small and makes the size of the prevalence in the age group of 75 plus grow.

In accordance with the findings of the present research in proportions, a higher percentage of physical disabilities occurred among men than among women. Similarly, according to the results of the prevalence, the occurrence of physical disabilities was higher among men than among women. In a research concerned with defining and estimating the prevalence of physical disabilities in Australia [**17**] the prevalence of the following was 3% among men and 4.6% among women: the main disabling condition plus severe or profound handicap, circulatory, respiratory, arthritis, other musculoskeletal neurological or physical disabilities [**17**]. In an investigation concerned with the categorized prevalence of physical disabilities with regard to age and sex carried out by Ethgen et al. [**16**], in all age groups, 51% of the physically disabled were female and 49% were male. In some of these groups including the 35-54, this percentage was higher among men than among women [**16**]. In another study regarding the measurement of the prevalence of disabilities by Mont [**18**], it was indicated that the prevalence value was higher in some age groups among women in Nicaragua than among men and vice versa [**18**]. The relative divergence of the findings of our research with those in literature could be first of all due to selection bias. In the two aforementioned studies, the use of samples might not have been well representative of the target population. However, in our research, the data used belonged to the whole number of the physically disabled population and can be indeed representative of the target population. Another reason for the diverging results could be the different causes of physical disability. Another reason can be the different categorization method used in this study since it took into account all types of physical disabilities. However, in the other studies they also included other problems such as hearing and vision impairment as well.

According to the results in proportion, a higher percentage of physical disabilities occurred among urban than rural residents. On the contrary, the results in prevalence indicated a higher occurrence among rural than among urban residents. In the literature reviewed by the researchers of the present study, no similar results and data were found to compare this finding. The divergence between the findings in proportion and prevalence could be justified as the following: proportion is related to the size of population. Since there is a bigger population living in cities in Iran, a higher percentage is expected to occur among the urban residents. On the other hand, the prevalence value is not related to the size of population. This way, the rural residents were found to be more prone to physical disabilities than the urban residents. The possible underlying causes could be jobs such as farming and animal raising which increase the probability of occurrence of physical injuries.

Our research indicated that the prevalence of physical disabilities in Iran during the recent years was approximately 8 per thousand people in the whole population. In a study by Hairi [**19**] concerning the prevalence of physical disabilities and the performance limitations among the aged population of Malaysia, the prevalence value was estimated to be 58% in the age group of above 60 among men and 124% among women of the same age group [**19**]. The great divergence of the results of this study and that of ours could be for the most part due to the fact that in the Malaysian study, the prevalence was only narrowed down to the aged group. This age group was found to have a similar prevalence of physical disabilities in our study as well (**Table 1**): about 55 in 2006 and about 37 per thousand people in 2011). The prevalence of disabilities in this group was observed to be much higher than that of the other age groups. However, in the Iranian study, the prevalence of disabilities in all the age groups was taken into account.

## Conclusion

The results of the present research are indicative of the prevalence trend of physical disabilities during 2006 and 2011 in the provinces of Iran. This information can be used to identify the high- and low-risk areas and can also help in better organizing the limited sources and facilities and fairly distributing them among the disabled. This information can be used as well in the etiology and finding the influential factors which cause the residents of particular areas to be more prone to physical disabilities than the others.
